# Anti-Angiogenic Effect of *Nelumbo nucifera* Leaf Extracts in Human Umbilical Vein Endothelial Cells with Antioxidant Potential

**DOI:** 10.1371/journal.pone.0118552

**Published:** 2015-02-25

**Authors:** Jong Suk Lee, Shruti Shukla, Jung-Ae Kim, Myunghee Kim

**Affiliations:** 1 Department of Food Science and Technology, Yeungnam University, Gyeongsan, Gyeongbuk, 712-749, Republic of Korea; 2 College of Pharmacy, Yeungnam University, Gyeongsan, Gyeongbuk, 712-749, Republic of Korea; Duke University Medical Center, UNITED STATES

## Abstract

*Nelumbo nucifera* Gaertn (Nymphaeaceae) has long been used as a traditional herb in Chinese, Japanese, Indian, and Korean medicinal practices since prehistoric times and flourishes today as the primary form of medicine. This study reports for the first time the potent ability of *N*. *nucifera* leaf extracts to inhibit vascular endothelial growth factor (VEGF)-induced angiogenesis *in vitro* and *in vivo*, as well as their antioxidant efficacy in various scavenging models and an analysis of their chemical composition. *In vivo* anti-angiogenic activity was evaluated in a chick chorioallantoic membrane (CAM) model using fertilized chicken eggs, in human umbilical vein endothelial cells (HUVECs) by using cell viability, cell proliferation and tube formation assays, and by determining intracellular reactive oxygen species (ROS) *in vitro*. The antioxidant efficacy of *N*. *nucifera* leaf extracts was determined in various scavenging models, including total phenolic and flavonoid content. The chemical composition of *N*. *nucifera* leaf extracts was determined by GC-MS analysis, which revealed the presence of different phytochemicals. The IC_50_ values for the DPPH radical scavenging activities of water and methanol extracts were found to be 1699.47 and 514.36 μg ml^−1^, and their total phenolic and flavonoid contents were 85.01 ± 2.32 and 147.63 ± 2.23 mg GAE g dry mass^−1^ and 35.38 ± 1.32 and 41.86 ± 1.07 mg QA g dry mass^−1^, respectively. *N*. *nucifera* leaf extracts (10–100 μg ml^−1^) exhibited significant dose-dependent inhibition of VEGF-induced angiogenesis, as well as VEGF-induced proliferation and tube formation in HUVECs. In this study, *N*. *nucifera* leaf extracts displayed potent antioxidant and inhibitory effects on VEGF-induced angiogenesis. *N*. *nucifera* exerted an inhibitory effect on VEGF-induced proliferation and tube formation, as well as CAM angiogenesis *in vivo*. Moreover, *N*. *nucifera* leaf extracts significantly blocked VEGF-induced ROS production in HUVECs, confirming their possible anti-angiogenic mechanism.

## Introduction

Herbal medicine consists of natural plant substances that have been used to prevent and treat ailments since ancient times. *Nelumbo nucifera* Gaertn (*N*. *nucifera*), commonly known as lotus, is a large aquatic plant and has long been used as a medicinal herb in China, Japan, India, and Korea [1]. In Ayurveda, this plant is used as a diuretic and anthelmintic, as well as in the treatment of strangury, vomiting, leprosy, skin diseases, and nervous exhaustion [2]. All parts of *N*. *nucifera*, including the leaves, flowers, embryos, and rhizomes, are prescribed as demulcents for hemorrhoids and are beneficial for the treatment of various human diseases [2].

*N*. *nucifera* leaves have recently gained popularity in Taiwan as an ingredient in health-related beverages for weight loss [1]. Several studies have shown that *N*. *nucifera* possesses pharmacologic and physiologic activities, including antioxidant [3], antiviral and immunomodulatory effects [4]. Recently, flavonoid-enriched *N*. *nucifera* leaf extracts were reported to inhibit the proliferation of breast cancer *in vitro* and *in vivo*, improve lipid metabolism, and relieve liver damage resulting from a high fat diet [1]. Moreover, the anti-obesity potential of *N*. *nucifera* leaves has been demonstrated via increased lipolysis in adipose tissue in mice [5]. Another report also indicated that *N*. *nucifera* leaves possess inhibitory activity towards atopic dermatitis [6].

A process of angiogenesis completes following several major steps, including proliferation, sprouting, elongation, and migration of endothelial cells [7]. However, abnormal and unregulated process of pathological angiogenesis may result in various pathogenesis diseases such as diabetic retinopathy, myocardial infraction, and chronic inflammation [7, 8, 9]. Hence, to control or inhibit the angiogenesis could be considered an important strategy in anti-cancer therapy.

The angiogenesis growth factors and their receptors play a key role in its regulation via tyrosine kinases [9]. Among the various angiogenic molecules, vascular endothelial growth factor (VEGF) is a major target for anti-angiogenic therapy [8]. Increased VEGF signaling in tumor and chronic inflammatory tissues induces endothelial cells to exit quiescence and undergo various angiogenic responses [8]. The VEGF plays an important role in binding and inducing the vascular endothelial growth factor receptor (VEGFR), which results in the auto-phosphorylation of tyrosine residues onVEGFR-2 receptor in endothelial cells [8, 9].

Additionally, VEGFR-2 through activating endothelial membrane NADPH oxidase results in the generation of reactive oxygen species (ROS) in response to various stress and growth stimuli [10, 11]. Since any type of inflammation can contribute to angiogenesis and tumor progression, plant-derived phytochemicals are emphasized as potential drugs to treat cancer and angiogenesis-related diseases [9].

Recently, investigations into new sources of natural antioxidants have become very important to improve human health. Plants are considered rich sources of polyphenol compounds such as flavonoids which exert potent antioxidant ability [3]. A number of plant-based polyphenols including flavonoids exhibit significant biological potential in terms of their remarkable antioxidant activities, as well as scavenge ROS and inhibit metal chelation and lipid-peroxidation [12]. Hence, phytochemicals can directly interfere with signaling systems involved in the regulation of angiogenesis and cancer invasion in a manner that is dependent on their antioxidant activity and concomitant inhibitory effect on protein kinases [13].

Although biological and therapeutic efficacies of *N*. *nucifera* have been reported to a certain extent, systematic studies on the anti-angiogenic effects of *N*. *nucifera* leaves have yet to be performed. Following traditional claims regarding the use of *N*. *nucifera* leaves to cure numerous diseases, in the present study, we investigated the ability of water and methanol extracts of *N*. *nucifera* leaves to inhibit VEGF-induced angiogenesis *in vitro* and *in vivo*, along with their antioxidant efficacy in various scavenging models. The chemical composition of both the extracts was also analyzed.

## Methods

### Chemicals and Cell Lines

The compounds, 2,2-diphenyl-1-picrylhydrazyl (DPPH), Folin-Ciocalteu's reagent, gallic acid, quercetin, cortisone acetate, VEGF, 3-(4,5-dimethylthiazol-2-yl)-2,5-diphenyltetrazolium bromide (MTT), 2,7-dichlorofluorescein diacetate (DCF-DA), trichloroacetic acid (TCA), dimethyl sulfoxide (DMSO), and sodium pyruvate, were purchased from Sigma-Aldrich (St. Louis, MO, USA). Fetal bovine serum (FBS), penicillin, and streptomycin were obtained from Gibco BRL (Grand Island, NY, USA). Human umbilical vein endothelial cells (HUVECs) and endothelial cell basal medium-2 (EBM-2) were purchased from Clonetics (San Diego, CA, USA). Endothelial cell growth media kit (EGM-2 Single-Quot) was purchased from Lonza (Walkersville, MD, USA). Matrigel basement membrane matrix was purchased from BD Biosciences (Bedford, MD, USA).

### Plant Material

*N*. *nucifera* leaves were collected in March 2012 from local lotus farm, located in Geumgang-dong, Dong-gu, Daegu city 701–330, Republic of Korea, and no specific permissions were required for these locations/activities. This study also did not involve any endangered or protected species. Moreover, no field study was carried out in this research; hence, no ethics statement is required for this study. Taxonomic identification of *N*. *nucifera* leaves was conducted by the herbarium incharge at the Department of Botany, Dr. H. S. Gour University, Sagar, MP, India. A voucher specimen (Bot/Her/1114) was deposited in the herbarium of the Laboratory of Microbiology, Department of Botany, Dr. H. S. Gour University, Sagar, MP, India.

### Preparation of Leaf Extracts

*N*. *nucifera* leaves were washed, cut, and stored at −20°C. For the preparation of water extracts, fresh whole leaves (100 g) were extracted with a 20-fold volume of distilled water for 3 h at 70°C. For the preparation of the methanol extract, 100 g of leaf sample was added to 1,000 ml of methanol, after which the mixture was stirred using a magnetic stirrer for 3 h. The extracts were then filtered through Whatman No. 2 filter paper (Maidstone, UK). The filtrates of the water and methanol extracts were concentrated using a vacuum evaporator, freeze-dried, and then stored at −20°C for further use.

### Total Phenolic Content

The total phenolic content was measured spectrophotometrically using the modified Folin-Ciocalteu colorimetric method [14]. Briefly, 20 μl of each diluted extract was added to 100 μl of Folin-Ciocalteu reagent. After 3 min, 80 μl of 10% aqueous sodium carbonate solution was added to the mixture. The solution was allowed to stand for 1 h at room temperature (RT), and the absorbance of the resulting blue-colored mixture was measured at 765 nm against a blank containing only the extraction solvent (200 μl). The amount of total phenolics was calculated as gallic acid equivalents (GAE) from the calibration curve obtained using a gallic acid standard solution and expressed as mg GAE g dry mass^−1^.

### Total Flavonoid Content

The total flavonoid content of the water and methanol leaf extracts of *N*. *nucifera* was determined by the colorimetric method [15]. Briefly, 100 μl of each extract or standard reagent and 400 μl of ethanol were mixed with 500 μl of a 2% solution of AlCl_3_ diluted in distilled water. After 1 h incubation at RT, the absorbance was measured at 430 nm. Quercetin was used to generate a standard curve, and the results were expressed as mg of quercetin equivalents (QE) g dry mass^−1^.

### Evaluation of Antioxidant Activity

#### DPPH Radical Scavenging Activity

DPPH assays were carried out as reported previously [16]. Briefly, different concentrations (100, 200, 500, and 1000 μg ml^−1^) of water and methanol extracts were mixed with 190 μl of 200 μM DPPH in methanol. After 30 min, the absorbance was measured at 517 nm using a micro-plate reader (Tecan, Mannedorf, Switzerland). Water and methanol solvents were used as a blank, while ascorbic acid was used as a positive control. DPPH radical scavenging ability was calculated using the following equation, in which *H* and *H*_*0*_ are the optical densities of the solvent with and without sample, respectively. Radical scavenging activity (%) = {(1–*H*)/*H*_*0*_} × 100

#### Reducing Power Ability

The reducing power of the water and methanol extracts was determined according to the method described previously [17]. Briefly, 1 ml of each extract sample or standard reagent (ascorbic acid as a positive control) at various concentrations (100, 200, 500, and 1000 μg ml^−1^) was mixed with 2.5 ml of 0.2 M sodium phosphate buffer (pH 6.6) and 2.5 ml of 1% (w/v) potassium ferricyanide solution. Later, the mixture was incubated at 50°C in a water bath for 30 min, mixed with 2.5 ml of 10% (w/v) TCA, and centrifuged at 3,000 x *g* for 10 min. Then, 250 μl of supernatant was mixed with 250 μl of DW, after which 500 μl of 0.1% (w/v) FeCl_3_ was added to the mixture. The absorbance was measured at 700 nm. Higher absorbance indicated greater reducing power. Water and methanol solvents were used as a blank, while ascorbic acid was used as a positive control.

#### Nitrite Radical Scavenging Activity

The nitrite radical scavenging activity of the water and methanol leaf extracts of *N*. *nucifera* was determined using the Griess reagent [18]. Briefly, 1 ml of each extract sample at various concentrations (100, 200, 500, and 1000 μg ml^−1^) was mixed with 1 ml of 1 mM NaNO_2_ solution. Then, 8 ml of 0.2 M citrate buffer (pH 3) was added to the mixture, followed by incubation for 1 h in a 37°C water bath. After incubation, 1 ml of the reaction mixture was added to a mixture of 2 ml of 2% (v/v) acetic acid and 0.4 ml of 1% (v/v) Griess reagent. This solution was then vigorously mixed and placed at RT for 15 min, after which the absorbance was measured at 520 nm. Water and methanol solvents were used as a blank, while ascorbic acid was used as a positive control. The scavenging activity of each sample or positive control was calculated by the following equation: Scavenging activity (%) = {1 − (Absorbance of treated sample—Absorbance of sample or control)/ Absorbance of control} ×100

### Evaluation of Anti-angiogenic Activity

#### Chick Chorioallantoic Membrane (CAM) Model

In this assay, the anti-angiogenic efficacy of the water and methanol leaf extracts of *N*. *nucifera* was evaluated according to a previously reported methods [8,9]. Fertilized chicken eggs were purchased from a local poultry farm and incubated at 37°C under 55% relative humidity. A hypodermic needle was used to make a small hole in the shell concealing the air sac. A second hole was made on the broad side of the egg directly over the vascular portion of the embryonic membrane, which was identified by candling. A false air sac was created beneath the second hole by applying negative pressure through the first hole, causing the CAM to separate from the shell. A window of approximately 1.0 cm^2^ was then cut into the shell over the dropped CAM using a small grinding wheel (Dremel, Racine, WI, USA). The window enabled direct access to the underlying CAM. To induce new blood vessel branches on the CAM of 10-d-old embryos, VEGF (2 μg ml^−1^) was used as a standard pro-angiogenic agent. Sterile discs (diameter: 10 mm) of Whatman No. 1 filter paper were pretreated with 3 mg ml^−1^ of cortisone acetate and air-dried under sterile conditions. Disks were suspended in 0.1 M phosphate buffered saline (PBS) containing VEGF, whereas control discs were suspended in 0.1 M PBS without VEGF, and then placed on growing CAMs. After 30 min, water and methanol leaf extracts of *N*. *nucifera* were added topically to the CAMs through the previously placed discs. The treated CAM samples were incubated for 3 d.

#### Microscopic Analysis

Microscopic analysis of CAM sections was performed according to a previously reported methods [8, 9]. Briefly, after 3 d of incubation at 37°C and 55% relative humidity, the tissues from the control and treated CAM samples beneath each filter disk were directly resected. Each tissue sample was washed three times with 0.1 M PBS, placed in a 35-mm diameter Petri dish (Nunc, Roskilde, Denmark), and then examined under a SV6 stereomicroscope (Zeiss, Oberkochen, Germany) at 50x magnification. Digital images of the CAM sections exposed to filters were collected using a three-charge-coupled device color video camera system (Toshiba, Kawasaki, Japan). The images were then analyzed using Image-Pro software (Media Cybernetics, Linton, UK). The numbers of vessel branch points contained in a circular region (equal to the area of each filter disk) were counted. One image was counted for each CAM preparation, and the findings from six to eight CAM preparations were analyzed for each treatment condition. The resulting angiogenesis index was measured as the mean ± standard deviation (SD) of the new branch points for each set of samples.

#### Cell Culture

HUVECs were grown in a 0.2% gelatin-coated flask and supplemented with EGM-2 Single-Quot, consisting of FBS, hydrocortisone, human fibroblast growth factor-B, VEGF, long R3 insulin-like growth factor-1, ascorbic acid, human epidermal growth factor, GA-1000, and heparin. HUVECs between passages 2 and 6 were used in the experiments.

#### Cell Viability Assay

The effect of water and methanol leaf extracts of *N*. *nucifera* on cell viability was assessed using the MTT staining method [8]. HUVECs from 4- to 5-d-old cultures were seeded in 96-well plates at a density of 2 x 10^4^ cells per well. For the control, cells were grown in the same medium containing sample-free vehicle. Cells were incubated with various concentrations of *N*. *nucifera* leaf extracts (1–1,000 μg ml^−1^) for 48 h. Then, 20 μl of MTT (5 g of MTT/l in 0.1 M PBS) was added, and the cells were incubated for an additional 4 h. Two hundred microliters of DMSO was added to each culture solution and mixed by pipetting to dissolve the reduced MTT crystals. Relative cell viability was determined by scanning at 540 nm on a micro-plate reader (Molecular Devices, Menlo Park, CA, USA).

#### Cell Proliferation Assay

Cell proliferation was measured according to a previously described method [8]. HUVECs coated at a density of 2 x 10^4^ cells per well on gelatin-coated 48-well micro-titer plates (Nunc, NY, USA) were incubated for 24 h in EBM-2 containing EGM-2 Single-Quots. After 24 h, the coated HUVECs were washed with EBM-2 and incubated for 6 h in EBM-2 containing only 1% (v/v) FBS. The coated HUVECs were then co-treated with various concentrations of water and methanol leaf extracts from *N*. *nucifera* and 20 ng ml^−1^ of VEGF solution. After incubation for 48 h, the numbers of viable cells were measured using the MTT assay.

#### Tube Formation Assay

Tube formation assay was performed using a 48-well micro-titer plate coated with 40 μl of Matrigel basement membrane matrix (BD Biosciences, Bedford, MD, USA) and incubated at 37°C for 30 min [8]. HUVECs were suspended in EBM-2 medium containing Glutamax and GA1000, plated on Matrigel at a density of 5 x 10^4^ cells per well and treated with various concentrations of water and methanol leaf extracts of *N*. *nucifera*. After 18 h, cells were photographed with a digital camera attached to an inverted fluorescence microscope (TE 2000-U, Nikon, Tokyo, Japan).

#### Intracellular ROS

Intracellular ROS generation was measured using the fluorescent dye DCF-DA as described previously [8]. Confluent cells were pretreated with various concentrations (10, 50, and 100 μg CAM^−1^) of water and methanol extracts from *N*. *nucifera* leaves for 3 h and then treated with 20 ng ml^−1^ of VEGF. After incubation for 5 min, cells were loaded with 5 μM DCF-DA for 15 min at 37°C and imaged on an inverted fluorescence microscope. The fluorescence intensity was determined by analyzing the captured images using Image-Pro Plus version 5.1 (Media Cybernetics, Silver Spring, MD, USA).

### Gas Chromatography-Mass Spectrometry (GC-MS) Analysis

A detailed analysis of the chemical composition of the water and methanol leaf extracts of *N*. *nucifera* was performed according to the method of Selvamangai and Bhaskar [19] using a GC/MS system (Jeol JMS 700 mass spectrometer, Agilent 6890N, Agilent Technologies, Santa Clara, CA, USA) equipped with a fused silica capillary column (30 m length× 0.25mm ID × 0.25 μm film thickness). GC-MS conditions were followed as reported previously [19]. The relative proportions of the extract constituents were expressed as percentages by peak area normalization. Extract components were identified based on GC retention time relative to computer matching of mass spectra using Wiley and National Institute of Standards and Technology Libraries for the GC-MS system.

### Statistical Analysis

The data were expressed as the mean ± SD of three independent experiments and analyzed using one-way analysis of variance and Student’s *t*-test. p values of <0.05 were considered to be statistically significant.

## Results and Discussion

### Total Phenolics, Flavonoids, and Antioxidant Activity

Selecting the proper extraction method is a very important parameter for obtaining extracts with acceptable yields and strong antioxidant capacity. Different extraction techniques separate soluble plant metabolites through the selective use of solvents. Therefore, the selection of a proper solvent may affect the quantity and quality of the resulting extracts. Polar solvents such as methanol can extract polar substances, whereas non-polar solvents can extract non-polar substances. Various organic compounds including phenolics and flavonoids have significantly higher solubility in methanol and water. Hence, methanol could be used to extract the majority of polar and non-polar compounds, whereas water is able to extract all highly polar compounds from different plant materials. Therefore, in this study, adequate methanol and water solvent systems were selected for extraction purposes, and the yields of water and methanol extracts of *N*. *nucifera* leaves were found to be 19.8% and 22.7% (w/w), respectively.

Phenolic compounds have beneficial biological effects to scavenge free radicals [20]. A number of studies conducted on plant samples in order to evaluate their antioxidant efficacy have confirmed that plant-based organic extracts rich in phenolic compounds exert potent antioxidant activities [20, 21]. The total phenolic content of *N*. *nucifera* leaf extracts was determined from a gallic acid standard curve and expressed as GAE g dry mass^−1^. The obtained values of the total phenolic content for the water and methanol leaf extracts of *N*. *nucifera* were 85.01 ± 2.32 mg GAE per g and 147.63 ± 2.23 mg GAE per g, respectively.

In human diet, flavonoids are found in plants ubiquitously, and known as the most commonly representatives of polyphenolic compounds. Among them, quercetin has shown great potential to exhibit anti-inflammatory and antioxidant properties [22, 23]. The content of flavonoid compounds in water and methanol extracts of *N*. *nucifera* leaves was determined using a standard calibration curve of quercetin and expressed as QE per g dry mass. The flavonoid content of water and methanol leaf extracts were found to be 35.38 ± 1.32 mg QE per g and 41.86 ± 1.07 mg QE per g, respectively.

In the DPPH assay, water and methanol leaf extracts of *N*. *nucifera* exhibited dose-dependent DPPH radical scavenging activities. Similarly, methanol extracts of *Camellia sinensis*, *Ficus bengalensis*, and *Ficus racemosa*, whose acetone extracts contain comparatively high levels of total phenolics, have been shown to display DPPH radical scavenging activities in a dose-dependent manner [24]. At various tested concentrations (100, 200, 500, and 1,000 μg ml^−1^), the DPPH scavenging activities of water and methanol leaf extracts of *N*. *nucifera* ranged from 0.59 ± 0.42% to 28.30 ± 1.13% and 16.84 ± 1.54% to 86.67 ± 0.20%, respectively (Table 1). However, in this assay, the IC_50_ values of the water and methanol leaf extracts were found to be 1699.47 μg ml^−1^ and 514.36 μg ml^−1^, respectively (Table 1). A higher DPPH radical scavenging activity is associated with a lower IC_50_ value. The highest concentration (1,000 μg ml^−1^) of the water and methanol leaf extracts of *N*. *nucifera*, as well as ascorbic acid (a positive control) at a concentration of 200 μg ml^−1^, showed DPPH radical scavenging activities by 28.30 ± 1.13%, 86.67 ± 0.20%, and 93.00 ± 0.24%, respectively (Table 1). It was confirmed in this study that the methanol extract showed better DPPH radical scavenging activity than the water extract. Furthermore, this phenomenon was also confirmed by the fact that the methanol leaf extract of *N*. *nucifera* had a higher total phenolic content compared to the water extract.

**Table 1:** DPPH and nitrite radical scavenging activities and reducing power of water and methanol extracts from *N*. *nucifera* leaves.

Sample (μg ml^−1^)	*Control (μg ml^−1^)	DPPH radical scavenging activity (%)			Nitrite radical scavenging activity (%)			Reducing power activity (absorbance at 700 nm)		
		Water extract	Methanol extract	Ascorbic acid	Water extract	Methanol extract	Ascorbic acid	Water extract	Methanol extract	Ascorbic acid
100	50	0.59±0.42	16.84±1.54	22.07±0.66	5.41±1.47	14.19±0.49	15.6±2.76	0.08±0.00	0.12±0.00	0.27±0.01
200	100	5.19±0.88	25.13±0.29	47.58±0.42	12.75±0.62	21.33±1.94	59.29±2.34	0.11±0.00	0.22±0.00	0.51±0.02
500	150	16.29±1.70	56.26±2.23	71.64±0.21	23.20±1.10	47.43±2.07	88.94±2.30	0.16±0.01	0.76±0.01	0.80±0.01
1000	200	28.30±1.13	86.67±0.20	93.00±0.24	43.42±1.14	54.76±1.84	97.05±0.54	0.20±0.02	0.89±0.01	1.06±0.02
** IC_50_ (μg/ml)		1699.47	514.36	106.48	1141.33	784.02	95.51	-	-	-

All data are expressed as the mean ± SD (n = 3).

*Ascorbic acid was used as a positive control for the DPPH radical scavenging, nitrite radical scavenging, and reducing power activity assays.

** IC_50_ (μg ml^−1^): concentration at which 50% of the activity is inhibited.

Since nitrite radicals are present abundantly in various protein-rich foods, meat, vegetables, medicine, and residual pesticides, they form nitrosamines upon reacting with amines present in these stuffs [21]. Nitrosamines can react with diazoalkanes, proteins, and intracellular components, resulting in an increased risk of cancer and other related disorders [25]. In this assay, the nitrite scavenging activity of the methanol leaf extract (54.76 ± 1.84%) was higher than that of the water leaf extract (43.42 ± 1.14%) (Table 1) at a concentration of 1,000 μg ml^−1^. The results showed that the percentage of inhibition was dose-dependent. Sreevidya et al. [26] reported the nitric oxide scavenging activity of hexane, ethyl acetate, and aqueous-alcohol extracts of *Chlorophytum tuberosum* containing sugars, saponins, and tannins, which showed potent antioxidant activity. In the present study, the concentrations of water and methanol leaf extracts needed for 50% inhibition (IC_50_) were found to be 1141.33 μg ml^−1^ and 784.02 μg ml^−1^, respectively, whereas a concentration of 95.51 μg ml^−1^ was needed for ascorbic acid (Table 1). The results were found to be statistically significant (p<0.05). The high nitrite scavenging activity of the methanol leaf extract may be due to the increased content of phenolic compounds, which occur naturally in plants, as also confirmed previously [18].

Transformation of Fe(III) to Fe(II) ions is considered a major step in determining the reducing power ability of any test compound, and reduction of Fe (III) ions results in the hydrogen atom release from the phenolic compound [21]. In this assay, *N*. *nucifera* leaf-derived water and methanol extracts showed dose-dependent Fe(III)-reducing ability, as indicated by their absorbance levels. Over the concentration range of 100 to 1,000 μg ml^−1^, the reducing power of the water and methanol leaf extracts increased from 0.08 to 0.20 and 0.12 to 0.89, respectively (Table 1). Moreover, a direct correlation between the antioxidant activity, radical scavenging activity and reducing power of certain plant extracts has also been observed previously [23].

### Cytotoxicity and Intracellular ROS

**Fig 1:**
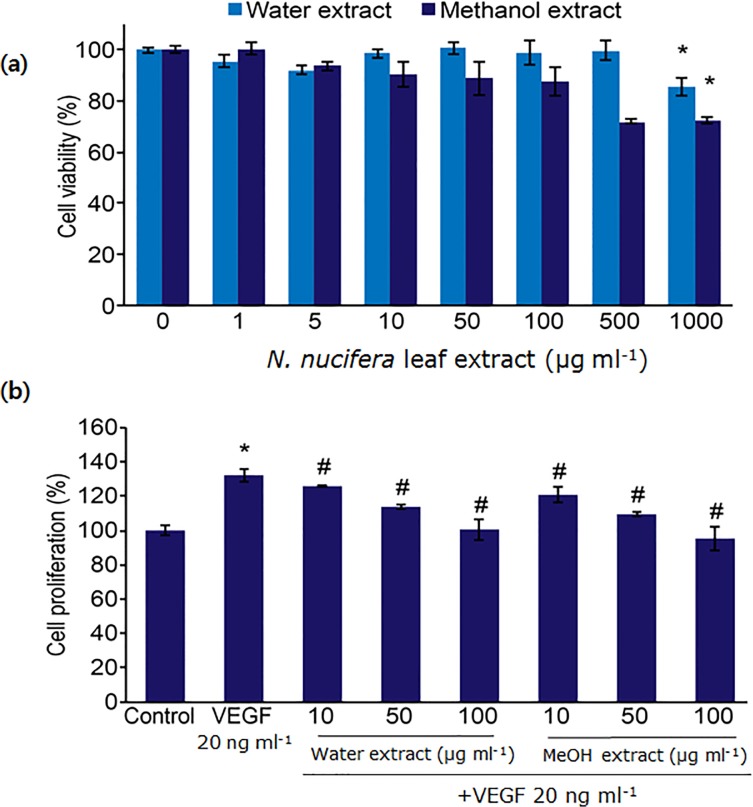
Effects of water and methanol extracts of *N*. *nucifera* leaves on cell viability and VEGF-induced proliferation. **(a)** Cell viability: HUVECs were treated with various concentrations of extracts. After 48 h, cell viability was measured using the MTT assay. *: p<0.05 compared with untreated control (0 μg ml^−1^). **(b)** VEGF-induced proliferation: HUVECs were co-treated with various concentrations of extracts and VEGF for 48 h. The number of viable cells was measured by using the MTT assay. *: p<0.05 compared with untreated control; ^#^: p<0.05 compared the VEGF-treated group. All tested concentrations (10, 50, and 100 μg ml^−1^) of the water as well as the methanol extracts displayed statistically significant differences with respect to each other.

The cytotoxic effects of *N*. *nucifera* leaf extracts were examined using the MTT assay to determine the effective concentrations required for the treatment. Exposing HUVECs to 1 to 1,000 μg ml^−1^ of the water extract for 48 h did not reduce cell viability; however, the viability of cells exposed to 500 and 1,000 μg ml^−1^ of the methanol extract for 48 h was reduced to 25% compared with the viability of control cells (Fig. 1A). These findings indicate that *N*. *nucifera* leaf extracts did not affect the viability of HUVEC cells at concentration lower than 100 μg ml^−1^ (Fig. 1A).

Many food-derived compounds display anti-angiogenic activity by targeting one or more steps in the angiogenesis signaling pathway [27], including the capsaicin-mediated inhibition of VEGF-induced tube formation in HUVECs and VEGF-induced molecular signaling pathway activation [27]. To determine the anti-angiogenic activity of *N*. *nucifera* leaf extracts *in vitro*, their inhibitory effects on the VEGF-induced proliferation of endothelial cells were examined. Treatment with *N*. *nucifera*-derived water and methanol leaf extracts (10, 50, and 100 μg ml^−1^) significantly inhibited VEGF-induced HUVEC proliferation in a concentration-dependent manner (Fig. 1B). At all tested concentrations (10, 50, and 100 μg ml^−1^), the water and methanol extracts showed statistically significant differences with respect to each other (Fig. 1B).

**Fig 2:**
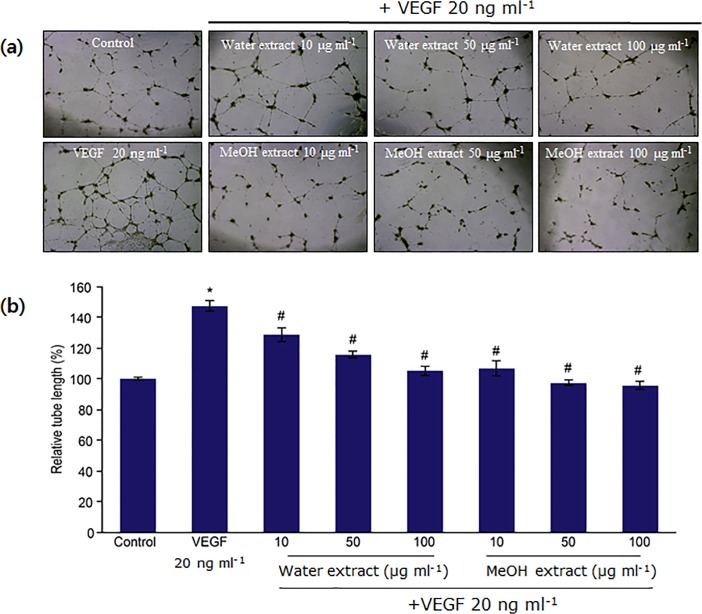
Inhibitory effects of water and methanol extracts of *N*. *nucifera* leaves on tube formation in HUVECs. **(a)** HUVECs (5 x 10^4^ cells) were plated on wells that had been previously coated with 40 μL of growth factor-reduced Matrigel basement membrane matrix. Cells were then treated with extracts in the presence of VEGF (20 ng ml^−1^). After 14 h, cells were photographed with a digital camera under a phase contrast microscope at 40x magnification. **(b)**The bar graph represents the relative area covered by the tube network, and data are shown as the mean ± SD. *: p<0.05 compared with untreated control; ^#^: p<0.05 compared with VEGF-treated group.

Furthermore, we examined the effects of both extracts on HUVEC tube formation. VEGF treatment significantly enhanced tube formation, which was inhibited by the water and methanol leaf extracts of *N*. *nucifera* in a dose-dependent manner (Fig. 2). According to the relevant literature, the inhibitory effects of many dietary polyphenols, including green tea polyphenols and mushroom polyphenols, on angiogenesis, metastasis, and tumor growth are believed to be mediated through the regulation of VEGFR-dependent signaling pathways [28]. Our results suggest that *N*. *nucifera* leaf extracts significantly blocked VEGF-induced angiogenesis *in vitro*. Moreover, the anti-angiogenic activities of the water and methanol leaf extracts were in accordance with their antioxidant activities, which may be attributed to the presence of large amounts of total phenolics and flavonoids in both the extracts.

**Fig 3:**
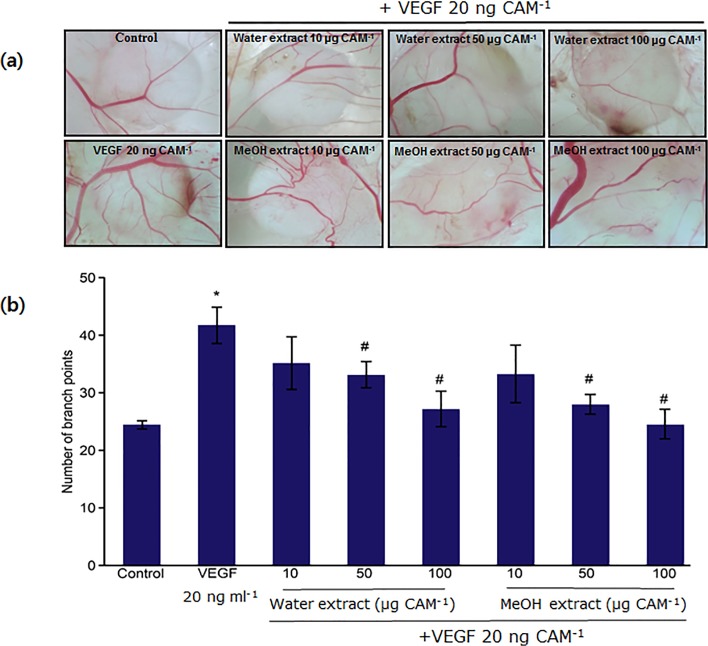
Inhibitory effects of water and methanol extracts of *N*. *nucifera* leaves on VEGF-induced angiogenesis. **(a)** The CAM of a 10-d-old chick embryo was separately exposed to PBS (control) and VEGF (20 ng ml^−1^) by means of filter disks. After 30 min, extracts were introduced on top of the CAMs. After 72 h of incubation, the CAM tissue directly beneath each filter disk was resected, and digital images of the CAM sections were captured. **(b)** The bar graph represents the number of new branches formed from existing blood vessels. Photographs were imported into an image software program to visualize the new vessel branch points. Data are shown as the mean ± SD. *: p<0.05 compared with untreated control; ^#^: p<0.05 compared with VEGF-treated CAM samples. None of the tested concentrations (10, 50, and 100 μg ml^−1^) of the water as well as the methanol extract display statistically significant differences with respect to each other.

The ability of the water and methanol extracts of *N*. *nucifera* leaves to inhibit angiogenesis *in vivo* was determined using the CAM assay. The numbers of blood vessel branch points significantly increased upon VEGF treatment (Fig. 3), compared with the PBS-treated control group (Fig. 3). However, treatment with both the water and methanol leaf extracts of *N*. *nucifera* significantly suppressed VEGF-induced angiogenesis in a dose-dependent manner (Fig. 3). In strong support of these results, it was previously found that alliin from garlic displayed inhibitory activity towards fibroblast growth factor-2-induced tube formation and *in vivo* CAM angiogenesis [29]. Alliin was also found to inhibit VEGF-induced angiogenesis in the CAM [29].

**Fig 4:**
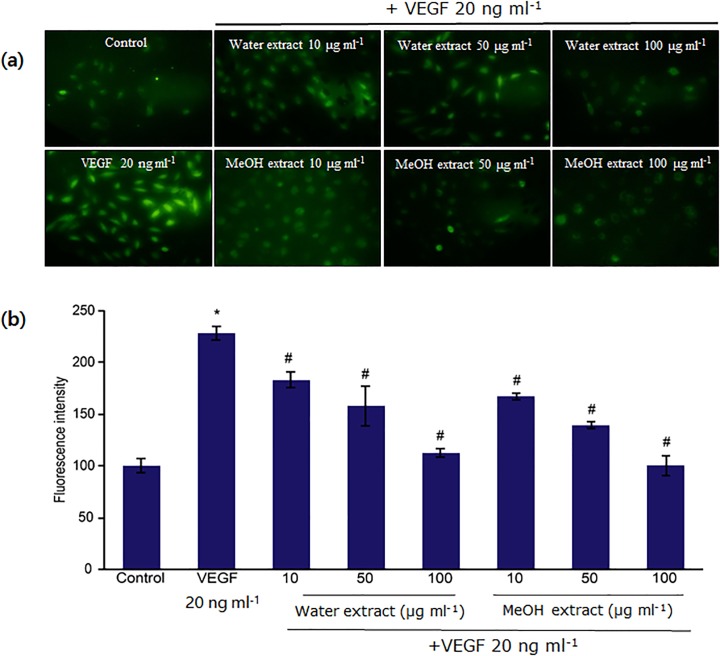
Inhibitory effects of water and methanol extracts of *N*. *nucifera* leaves on VEGF-induced ROS generation in HUVECs. **(a)** Serum-starved cells were pretreated with extracts for 30 min and then treated with VEGF (20 ng ml^−1^) for 15 min. ROS generation was determined by measuring DCF-DA fluorescence. The fluorescence intensity was determined by analyzing the captured images with the Image Inside program. **(b)** Data are mean ± SD values from four independent experiments. *: p<0.05 compared with untreated control; ^#^: p<0.05 compared with VEGF-treated CAM samples. All tested concentrations (10, 50, and 100 μg ml^−1^) of the water as well as the methanol extract display statistically significant difference with respect to each other.

Since ROS derived from growth factor-stimulated receptors are critically important in many aspects of vascular cell behavior including VEGF-induced angiogenesis [10], we examined whether *N*. *nucifera* leaf extracts inhibit VEGF-induced ROS production in HUVECs or not. Cells stimulated with VEGF showed intracellular ROS elevation as evidenced by cell labeling using the fluorescent probe DCF-DA (Fig. 4). During angiogenesis, a large increase in oxygen uptake resulted in the massive release of intracellular ROS in VEGF-treated cells (Fig. 4), compared with untreated control cells (Fig. 4). A significant increase in ROS production over basal levels was observed in VEGF-induced HUVECs (Fig. 4). However, pre-treatment with water and methanol leaf extracts significantly inhibited VEGF-stimulated intracellular ROS generation (Fig. 4).

### Chemical Composition Analysis

GC-MS analyses of the water and methanol leaf extracts from *N*. *nucifera* identified 10 and 9 different components, representing 99.98% and 98.44% of the total extracts, respectively. The leaf extracts of *N*. *nucifera* yielded compounds largely composed of hydrocarbons, alcohols, phenolics, alkaloids, and flavonones, as well as hydrazine and imidazole derivatives and some other essential phytochemicals. The profile of the detailed chemical composition analysis of the water and methanol leaf extracts of *N*. *nucifera* is summarized in Table 2.

**Table 2:** GC-MS chemical composition analysis of the water and methanol extracts of *N*. *nucifera* leaves.

No	Water extract			Methanol extract			Identification method^d^
	RT^a^	Compound^b^	Composition (%)^c^	RT^a^	Compound^b^	Composition (%)^c^	
1	1.77	Alaninol	72.46	1.77	N-Methyl Carbamate/ carbamic acid	79.06	EI-MS
2	2.39	Vinegar acid/Acetic acid	14.58	2.33	Vinegar acid/ Acetic acid	8.97	EI-MS
3	2.67	Butanedioic acid	0.91	2.72	Ethylic acid	3.93	EI-MS
4	2.79	Luprosil	3.92	3.61	Butyric acid	2.45	EI-MS
5	3.18	Peracetic acid	1.10	5.78	Pyrazine	0.20	EI-MS
6	3.16	Butyric acid	5.73	39.73	N- Methylasimilobine	1.57	EI-MS
7	5.80	Furanone	0.30	40.26	Azaphenanthrene	1.56	EI-MS
8	39.73	Methylasimilobine	0.48	41.78	Roemerine	0.48	EI-MS
9	40.26	Azaphenanthrene	0.35	46.52	Pyrazoline	0.22	EI-MS
10	41.78	Roemerine	0.15				EI-MS

^a^ Retention time (RT)

^b^ Compounds listed in order of elution from a DB-5 capillary column

^c^ Percentage based on FID peak area normalization

^d^ Identification based on computer matching of electron ionization mass spectra using Wiley and NIST Libraries for the GC-MS system.

Based on these analyses, reomerine was found to be present in both the water and methanol leaf extracts of *N*. *nucifera* (Table 2). Previously, Xu et al. [30] reported the presence of the structurally similar alkaloids nuciferine and roemerine in crude lotus leaf extract. Ahn et al. [31] reported a reomerine alkaloid from *N*. *nucifera* with anti-obesity effects. Rashid et al. [32] characterized alaninol, a flavone derivative from *Albizia lebbeck* with antimicrobial and antioxidant activities, which was also present in the water extract of *N*. *nucifera* leaves. Another important alkaloid, N-methylasimilobine, which is present in the methanol leaf extract of *N*. *nucifera*, has been reported to possess acetylcholinesterase inhibitory activity [33]. In addition, both the water and methanol leaf extracts contained a green pigment component, azaphenanthrene, that is of considerable importance to the food and pharmaceutical industries [34]. On the other hand, furanone, a phenolic compound that has been found to possess antioxidant and anti-inflammatory activities [35], was also present in the water extract. The easy oxidation of furanones in general, which is likely to be an important property for their role as signaling molecules, results in both anti-mutagenic and anti-carcinogenic activities [35]. Moreover, some furanones are found in fruits with reported antioxidant and anti-inflammatory activities [36]. Some of the other volatile organic acids present in both the water and methanol leaf extracts, such as vinegar acid, acetic acid, butyric acid, peracetic acid, and butanoic acid, can make an important contribution to the flavor characteristics of various foods while serving as antioxidant agents [37].

The major compound present in the methanol leaf extract of *N*. *nucifera* was N-methyl carbamate, a derivative of carbamic acid with reported insecticidal potential [38]. Other compounds present in methanol leaf extract were pyrazine derivatives, which comprise an important class of aromatic fragrances and are relevant components of the aromas of many fruits, vegetables, wines, and natural products [39]. Derivatives of pyrazine also exhibit potent ability to inhibit cyclooxygenase-enzyme, as well as show cardiovascular relaxing, anti-thrombotic, analgesic, and anti-aggregation properties [39, 40]. It has been reported that derivatives of imidazo [1,2-a] significantly decreased the expression of glycoprotein (GP) in Dami cells [40]. Moreover, some other pyrazine derivatives were found to enhance GPIIb/IIIa and inhibited the proliferation of human erythroleukemia cell line [40]. It is speculated that the potent antioxidant and anti-angiogenic effects of the water and methanol leaf extracts of *N*. *nucifera* might be correlated with the presence of various biologically active polyphenolic compounds in both extracts, which act either individually or in combination with profound synergistic effects. Angiogenesis is a multistep process, and oxidative damage can lead to the development of tumors through several distinct pathways. The polyphenols present in plants have been shown to regulate cell proliferation and induce apoptosis due to their versatile antioxidant potential [41].

### Common and Unusual Features

Experiments were carried out in fertilized eggs to investigate the effect of our plant samples on developing blood vessels. In this study, special care may have required during the handling of fertilized eggs. Another possible source of mishandling was in the preparation of the air sac at the central portion of the egg. The air sac is normally present in the corner side of the egg; however, properly checking the numbers of vessel branch points requires carefully moving this air sac into the central portion of the egg rather than the corner portion. Furthermore, at the time of well cutting and sample loading in the CAM, there is a possibility of breakage of the inner membrane of the egg rather than the outer membrane, and due to high pressure of the cutting machine, wells are also sometimes cut in the wrong direction; hence, in such cases, these eggs must be discarded to avoid misinterpretation of the results. We believe that it is also important to carefully use covering tape after loading the sample into the CAM wells.

### Additional Questions for Study

We previously examined the anti-angiogenic and anti-tumor activities of synthetic phenylpropenone derivatives through the inhibition of receptor tyrosine kinase [8]. These anti-angiogenic effects are consistent with the results of the present study. However, in the present study, we did not explore the detailed effects of the extracts to demonstrate their anti-angiogenic effects in a way that may or may not occur in cases attributed to other samples. These features include early effects on tube formation and branch points during the incubation periods. More detailed experiments, including receptor tyrosine kinase activity assays would greatly help to provide a better scientific understanding of these extracts.

### Conclusions

In conclusion, this study provides the first convincing and integrated evidence that *N*. *nucifera* leaf extracts have health-protecting effects as confirmed by their potent antioxidant effects and inhibitory activity towards VEGF-induced angiogenesis. Specifically, both the water and methanol leaf extracts of *N*. *nucifera* were found to be strong sources of total phenolic and flavonoid content and showed potent antioxidant effects in terms of their reducing power and with a significant capacity to scavenge DPPH and nitrite radicals. In addition, both the extracts displayed inhibitory activities towards VEGF-induced proliferation and tube formation as well as CAM angiogenesis *in vivo*. Moreover, this study demonstrated a possible molecular mechanism by which *N*. *nucifera* leaf extracts containing various antioxidant-rich phytochemicals inhibit VEGF-induced angiogenesis through the suppression of VEGF-induced ROS production. The phytoconstituents identified in the water and methanol extracts of *N*. *nucifera* leaves were found to be biologically active, thus confirming their role in preventing severe infections arising from microbial pathogenicity and oxidative stress caused by the over production of free radicals. Hence, we conclude that water and methanol extracts derived from *N*. *nucifera* leaves can be used as easily accessible sources of natural antioxidants for potential preventative therapies against angiogenesis-related diseases. However, further studies to identify the individual bioactive compounds present in *N*. *nucifera* leaves are planned in order to elucidate their precise mechanisms.
